# Oncolytic measles virus enhances antitumour responses of adoptive CD8^+^NKG2D^+^ cells in hepatocellular carcinoma treatment

**DOI:** 10.1038/s41598-017-05500-z

**Published:** 2017-07-12

**Authors:** Aiping Chen, Yonghui Zhang, Gang Meng, Dengxu Jiang, Hailin Zhang, Meihong Zheng, Mao Xia, Aiqin Jiang, Junhua Wu, Christian Beltinger, Jiwu Wei

**Affiliations:** 1 Jiangsu Key Laboratory of Molecular Medicine, Medical School, Nanjing University, Nanjing, 210093 China; 2 Nanjing University Hightech Institute at Suzhou, Suzhou, 215123 China; 3 Department of Pediatrics and Adolescent Medicine, University Medical Center Ulm, Ulm, 89075 Germany

## Abstract

There is an urgent need for novel effective treatment for hepatocellular carcinoma (HCC). Oncolytic viruses (OVs) not only directly lyse malignant cells, but also induce potent antitumour immune responses. The potency and precise mechanisms of antitumour immune activation by attenuated measles virus remain unclear. In this study, we investigated the potency of the measles virus vaccine strain Edmonston (MV-Edm) in improving adoptive CD8^+^NKG2D^+^ cells for HCC treatment. We show that MV-Edm-infected HCC enhanced the antitumour activity of CD8^+^NKG2D^+^ cells, mediated by at least three distinct mechanisms. First, MV-Edm infection compelled HCC cells to express the specific NKG2D ligands MICA/B, which may contribute to the activation of CD8^+^NKG2D^+^ cells. Second, MV-Edm-infected HCC cells stimulated CD8^+^NKG2D^+^ cells to express high level of FasL resulting in enhanced induction of apoptosis. Third, intratumoural administration of MV-Edm enhanced infiltration of intravenously injected CD8^+^NKG2D^+^ cells. Moreover, we found that MV-Edm and adoptive CD8^+^NKG2D^+^ cells, either administered alone or combined, upregulated the immune suppressive enzyme indoleamine 2,3-dioxygenase 1 (IDO1) in HCC. Elimination of IDO1 by fludarabine enhanced antitumour responses. Taken together, our data provide a novel and clinically relevant strategy for treatment of HCC.

## Introduction

Novel effective approaches are urgently required for treatment of hepatocellular carcinoma (HCC). Oncolytic viruses (OVs) are naturally occurring or genetically modified viruses that selectively replicate in and lyse tumour cells^1^. The most exciting findings in OV-mediated cancer therapies are their excellent capabilities in eliciting antitumour immune response^2^. A number of recent studies demonstrate that antitumour immunity plays a critical role in the overall efficacy of oncolytic virotherapy^3^. To achieve optimal antitumour immunity, OVs have been genetically modified to express tumour associated antigens (such as NY-ESO-1 or PSA) to prime and boost specific antitumour immune responses^4–6^, or to express cytokines (e.g. GM-CSF, IL-15) to augment activation of immune cells^7–9^. OVs have also been combined with other therapeutics to enhance antitumour immune responses, such as blockade of the immune checkpoints PD1/PDL1 and CTLA4^10, 11^, or with other immunotherapies^12^. The approval of oncolytic virus HSV-1 expressing GM-CSF (T-VEC) by the FDA is a recent milestone of viro-immunotherapy^13^.

Measles virus vaccine strain (MV) has been recognized to target multiple tumour entities, and are investigated in phase I/II clinical trials of recurrent glioblastoma (NCT00390299), ovarian carcinoma (NCT00408590, NCT02068794), multiple myeloma (NCT02192775, NCT00450814) and mesothelioma (NCT01503177)^3, 14–16^. Several preclinical studies have suggested that MV induces antitumour immunity. An *ex vivo* study showed that MV-infected mesothelioma cells promotes maturation of dendritic cells, inducing proliferation of tumour-specific CD8 T cells^17^. Intratumoural injection of MV expressing interferon-β enhanced infiltration of CD8 positive immune cells into mesotheliomas tumour^18^. Arming MV with granulocyte macrophage colony-stimulating factor (GM-CSF) improved T cell-mediated antitumour responses^19^. However, little is known about the mechanisms underlying MV-induced antitumour immune responses.

Adoptive cell transfer immunotherapy is an emerging approach to cancer treatment includes adoptive T cells, CAR T cells and cytokine induced killer (CIK) cells^20, 21^. CD8^+^NKG2D^+^ cells are a subpopulation of cytokine-induced killer cells, which present phenotypic and functional properties of both natural killer (NK) and T cells, and have MHC-independent antitumour activity both in solid tumours and hematologic malignancies^22–25^.

Indoleamine 2,3-dioxygenase 1 (IDO1) catabolizes tryptophan to kynurenine and has immunosuppressive roles in cancer^26^. In tumours, IDO1 can be induced by antitumour immunotherapy^27^. IFNs are potent inducers of IDO1 and other factors including IL-10 and TGF-β1 also induce IDO1^28^. Thus, induced IDO1 may counter-regulate antitumour immune responses by means of immunotherapy including viro-immunotherapy. However, it is yet unknown whether IDO1 play a role in OV-mediated antitumour immunity.

We set out to explore a novel and clinically relevant strategy for HCC treatment. To this end, we determined the antitumour efficacy of MV combined with adoptive transfer of CD8^+^NKG2D^+^ cells and investigated the associated mechanisms in HCC. Finally, we delineated the role of IDO1 in oncolytic viro-immunotherapy. Taken together, the results suggest a promising novel approach to the therapy of HCC warranting further study.

## Results

### MV-Edm infection in HCC cells augments CD8^+^NKG2D^+^-mediated antitumour efficacy

**Figure 1:**
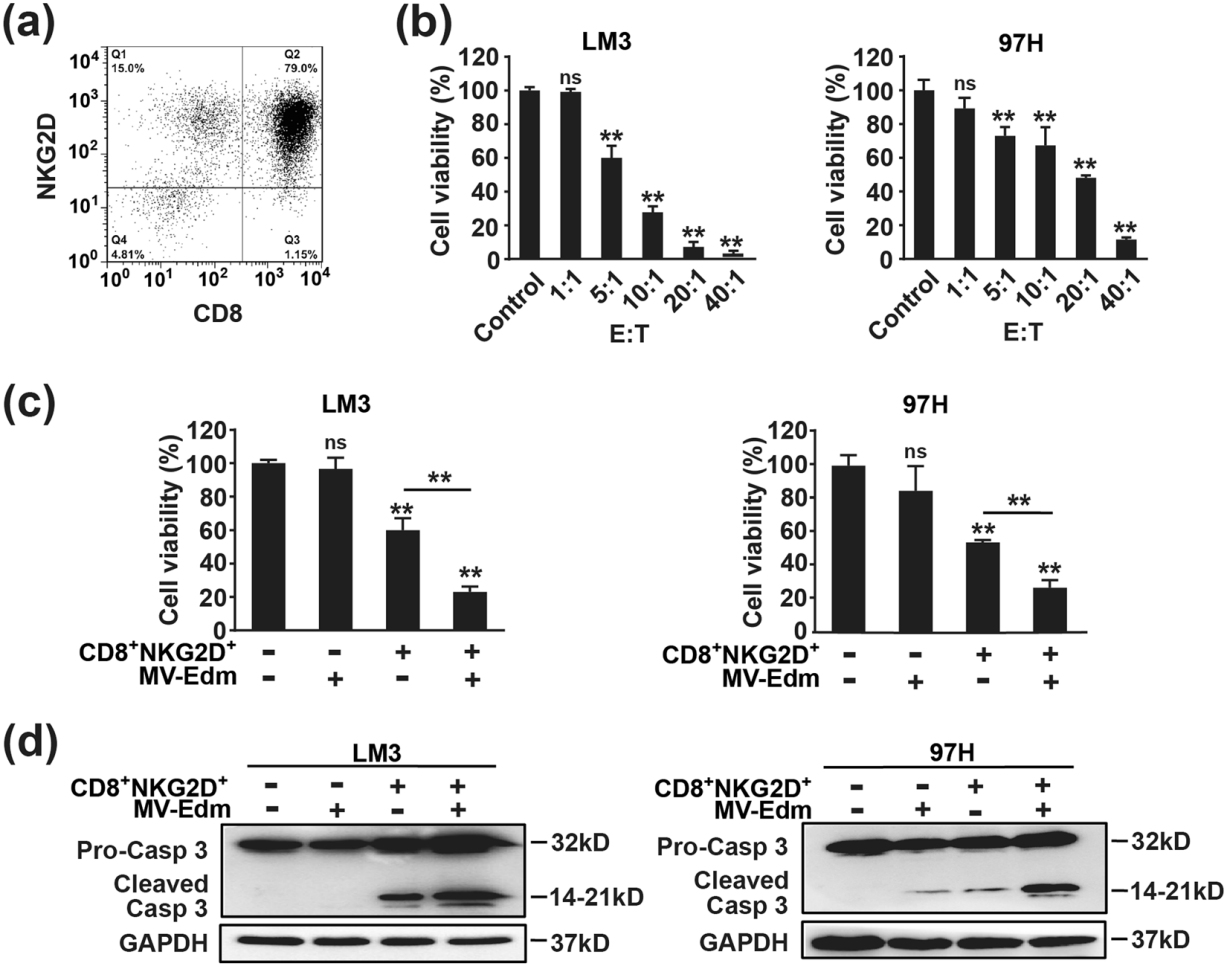
MV-Edm improves the killing activity of CD8^+^NKG2D^+^ cells *in vitro*. (**a**) PBMCs obtained from healthy donor were stimulated and cultured *ex vivo* as described in methods. 14 days later, cells were identified by flow cytometry using anti-CD3, anti-CD8 and anti-NKG2D antibodies. A representative identification of expanded cells is shown. (**b**) Hepatocellular carcinoma cell lines LM3 and 97H expressing luciferase were seeded in 96-well plates for overnight, then CD8^+^NKG2D^+^ cells were added into each well at a ratio (E:T) of 1:1, 5:1, 10:1, 20:1, and 40:1. 8 h later, luciferin was added and the plates were subjected to Luminescence spectrometry. Untreated HCC cells were used as controls. Means + SD of three independent experiments are shown. (**c**) LM3 and 97H cells expressing luciferase were infected with or without MV-Edm (MOI = 1). 24 h later, cells were further incubated with CD8^+^NKG2D^+^ cells for 8 h at a ratio (E:T) of 5:1 (left panel) and 10:1 (right panel), respectively. Then the cell viability was determined as described in (**b**). Untreated HCC cells were used as controls. Means + SD from three independent experiments are shown. (**d**) LM3 and 97H cells were infected with MV-Edm at a MOI of 1 for 24 h, then CD8^+^NKG2D^+^ cells were added at a ratio (E:T) of 2:1 for another 24 h. Cell lysates were then harvested for Western blot. Representative blots from three independent experiments are shown. *ns*, not significant; ** *P* < 0.01.

To investigate the capability of antitumour immune activation by MV-Edm, we generated *in vitro* a bulk cell population consisting of CD8^+^NKG2D^+^ (about 79%) from human peripheral blood mononuclear cells (Fig. 1a). Then we confirmed that CD8^+^NKG2D^+^ cells exerted well oncolysis when mixed with HCC cells at a ratio (E:T) over 5 to 1 (Fig. 1b). Next, we found that MV-Edm-infected HCC cells were more sensitive to CD8^+^NKG2D^+^-mediated oncolysis (Fig. 1c). Of note, at the time of cell death determination, MV-Edm alone had no significant cytotoxicity on HCC cells (Fig. 1c), indicating that the enhanced antitumour effect was mainly contributed by CD8^+^NKG2D^+^ cells. In line, cleaved form of caspase 3 was massively increased in MV-Edm-infected HCC cells followed by CD8^+^NKG2D^+^ treatment (Fig. 1d). These data suggests that MV-Edm infection of HCC cells significantly enhances CD8^+^NKG2D^+^-mediated antitumour efficacy.

### MV-Edm infection in HCC cells enhances antitumour immune activities of CD8^+^NKG2D^+^ cells ***in vitro***

**Figure 2:**
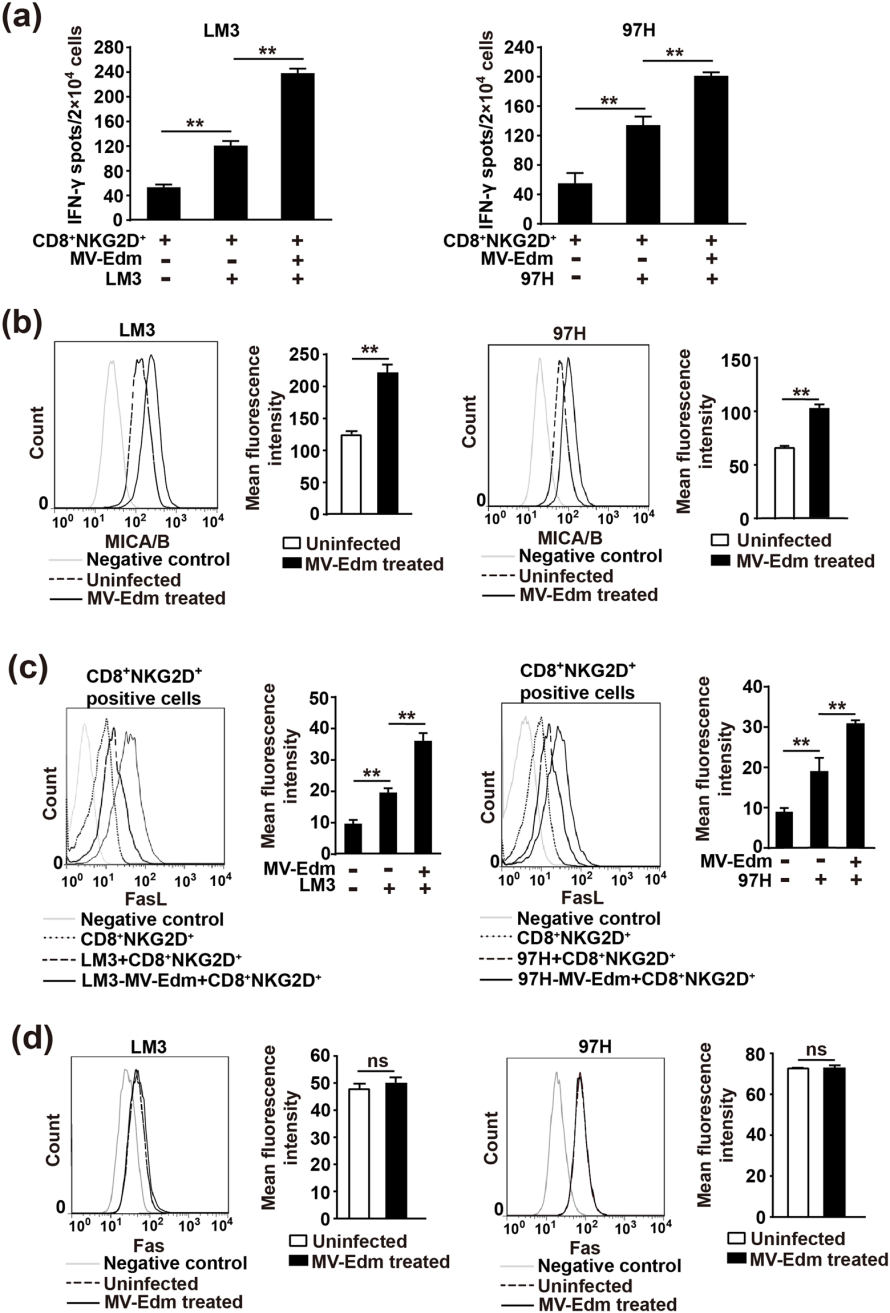
MV-Edm improves antitumour immune responses of CD8^+^NKG2D^+^ cells against HCC cells. (**a**) LM3 or 97H cells were infected with MV-Edm (MOI = 1) for 24 h or were left uninfected, then cells were harvested and mixed with CD8^+^NKG2D^+^ cells at a ratio of 2:1 (E:T) for 12 h. The number of IFN-γ-producing CD8^+^NKG2D^+^ cells was determined by IFN-γ ELISPOT assay kit. Means + SD of triplicate from two independent experiments are shown. **(b)** LM3 and 97H cells were infected with MV-Edm (MOI = 1) for 24 h, then cells were stained by anti-MICA/B-PE before subjected to flow cytometry. Unstained cells were used as negative controls. An overlay of histograms representative of 3 independent experiments, and mean fluorescence intensity of MICA/B averaged from 3 independent experiments are shown. (**c**) LM3 and 97H cells were infected with MV-Edm at a MOI of 1 for 24 h, then CD8^+^NKG2D^+^ cells were added at a ratio of 2:1 (E:T) for another 24 h. CD8^+^NKG2D^+^ cells were then harvested and stained with CD3-FITC and FasL-PE before subjected to flow cytometry. Fluorescence intensity of FasL was determined in CD3^+^ cells. Unstained cells were used as negative controls. An overlay of histograms representative of 3 independent experiments, and the mean fluorescence intensity of FasL averaged from 3 independent experiments are shown. (**d**) LM3 and 97H cells were infected with MV-Edm (MOI = 1) for 24 and 48 h, then cells were stained by anti-Fas-FITC before subjected to flow cytometry. Unstained cells were used as negative controls. An overlay of histograms representative of 3 independent experiments, and mean fluorescence intensity of MICA/B averaged from 3 independent experiments are shown. *ns*, not significant, ** *P* < 0.01.

Then we sought to clarify why MV-Edm infection augmented the antitumour responses of CD8^+^NKG2D^+^ cells. Using IFN-γ as a marker of antitumour immune activation, we found that the number of IFN-γ-producing CD8^+^NKG2D^+^ cells was stimulated by HCC cells and was further significantly increased by MV-infected HCC cells (Fig. 2a). We postulated that CD8^+^NKG2D^+^ cells could be directly activated via their activation receptor NKG2D, and thus we determined the expression of NKG2D ligands MICA/B in HCC cells. We found that MV-Edm infection significantly upregulated MICA/B in HCC cells (Fig. 2b). Interestingly, we found that MV-Edm-infected HCC cells increased FasL expression in CD8^+^NKG2D^+^ cells (Fig. 2c), which might strengthen their antitumour efficacy via Fas-mediated apoptotic signaling. However, MV-Edm infection did not affect the Fas expression in HCC cells (Fig. 2d). These data suggest that the improved antitumour immune responses of CD8^+^NKG2D^+^ cells are mediated in part by both increased MICA/B in MV-Edm-infected HCCs and increased FasL in CD8^+^NKG2D^+^ cells induced by MV-Edm-infected HCC cells.

It has been reported that MV-Edm induces immunogenic cell death (ICD) in melanoma cells^29^, and ICD plays a crucial role in inducing tumour antigen cross-presentation^30^. We therefore also determined the induction of ICD in HCC cells infected by MV-Edm. Indeed, MV-Edm infection promoted membrane translocation of calreticulin, a critical molecule in activating dendritic cells (Supplementary Fig. S1a). Moreover, the danger signals ATP and HMGB1, which are indispensable for innate and adaptive immune activation, were also significantly increased in supernatants from MV-Edm-infected HCC cells (Supplementary Fig. S1b,c).

Taken together, our data suggest that MV-Edm infection in HCC cells not only promotes antitumour immune activities of CD8^+^NKG2D^+^ cells, but may also play roles in adaptive antitumour immunity via induction of ICD.

### Local injection of MV-Edm into subcutaneous HCC improves CD8^+^NKG2D^+^-mediated therapeutic outcomes

**Figure 3:**
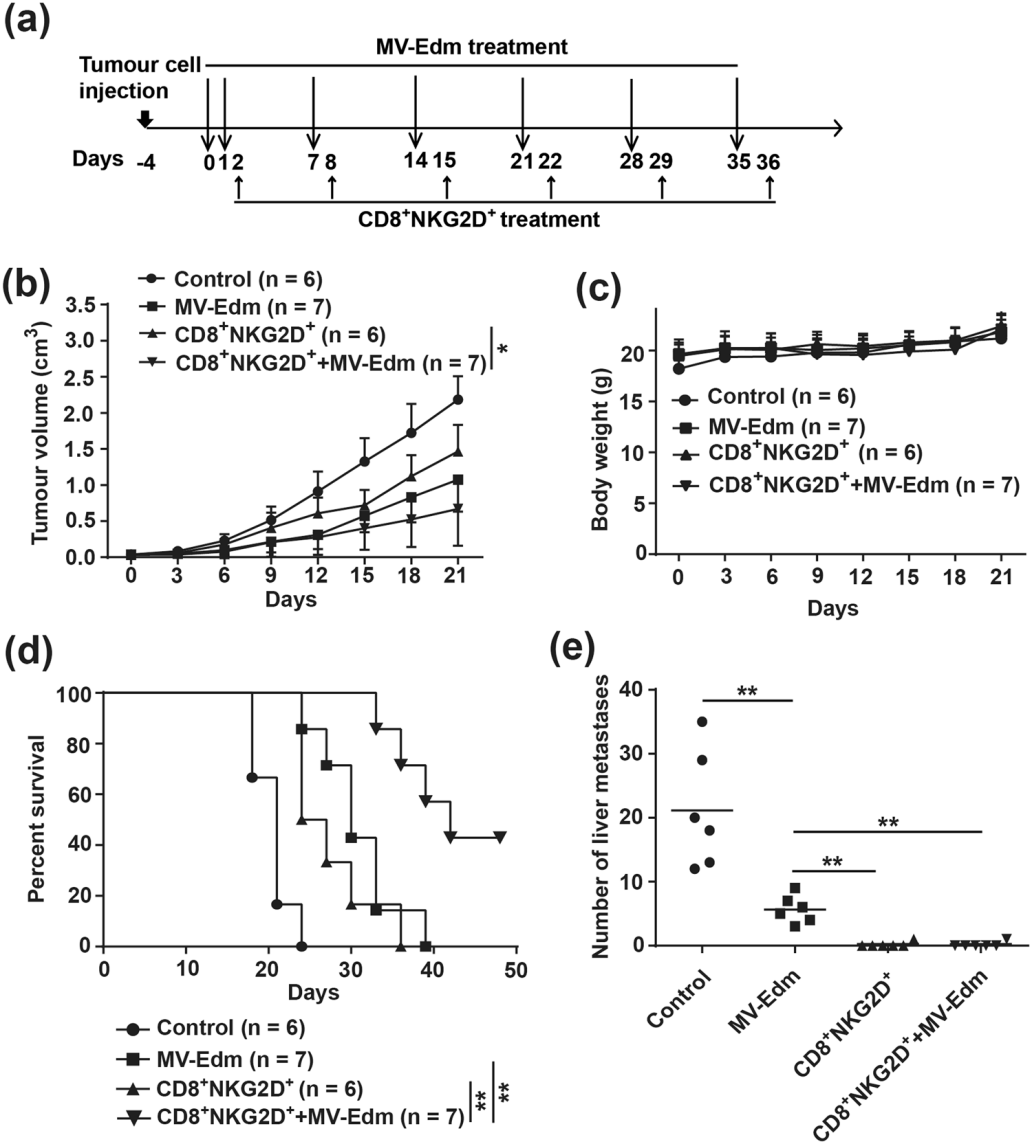
Combined therapies with MV-Edm and CD8^+^NKG2D^+^ cells achieve superior antitumour outcomes *in vivo*. 4- to 6-week-old male Balb/c nude mice received subcutaneous injection of 1 × 10^7^ LM3 cells in the right flanks. When tumours reached to an average volume of 40 mm^3^, mice were randomized to four groups. The mice received intratumoural injection of MV-Edm (5 × 10^6^ PFU per mouse each injection) on day 0, 1, 7, 14, 21, 28, 35 (filled squares, n = 7), or injected with CD8^+^NKG2D^+^ cells (1 × 10^7^ per mouse each injection) via tail vein once a week on day 2, 8, 15, 22, 29 and 36 (filled triangles, n = 6), or received intratumoural injection of MV-Edm followed by intravenous injection of CD8^+^NKG2D^+^ cells (filled inverted triangles, n = 7), or left untreated (filled circles, n = 6). Mice were sacrificed when tumour volume was over 2 cm^3^, or when mice appeared moribund. (**a**) A scheme depicts the schedules of HCC cells injection and the following treatments. **(b)** Tumour growth was measured by caliper, and **(c)** body weight was monitored every 3 days during the treatment. Means + SD of each group are shown. **(d)** Survival was determined and plotted for Kaplan-Meier survival analysis and analyzed by log-rank test. **(e)** Remote metastases were detected in multiple organs when mice died. Metastases were found only in the liver and the number of metastatic nodes was counted. * *P* < 0.05, ** *P* < 0.01.

Having shown that MV-Edm infection sensitized HCC cells to CD8^+^NKG2D^+^-mediated oncolysis and that MV-Edm-infected HCC cells improved antitumour immune activation of CD8^+^NKG2D^+^ cells, we next wanted to know if MV-Edm could improve CD8^+^NKG2D^+^-mediated antitumour efficacy *in vivo*. To this end, we established a subcutaneous HCC-bearing mouse model. The schema shows the therapeutic cohort (Fig. 3a). Intratumoural injection of MV-Edm enhanced CD8^+^NKG2D^+^-mediated antitumour efficacy and significantly inhibited HCC growth (Fig. 3b). No therapy-induced side effect was observed and no body weight lost occurred during treatment (Fig. 3c). Intratumoural injection of MV-Edm followed by intravenous transfer of CD8^+^NKG2D^+^ cells resulted in significantly prolonged survival (Fig. 3d). Interestingly, while all mice in untreated and MV-Edm-treated groups developed disseminated liver metastases, no liver metastases were detected in 5 out of 6 mice treated either CD8^+^NKG2D^+^ alone or MV-Edm combined with CD8^+^NKG2D^+^ cells, and only single metastasis was found in 1 out of 6 mice of each group (Fig. 3e). In our experimental HCC model, we did not observe any metastases outside the liver. Our data indicate that intratumoural injection of MV-Edm can efficiently improve therapeutic outcomes of adoptive transfer of CD8^+^NKG2D^+^ cells *in vivo*, and that CD8^+^NKG2D^+^ shows promise for controlling metastasis.

### Intratumoural injection of MV-Edm improves activation and infiltration of CD8^+^NKG2D^+^ cells

**Figure 4:**
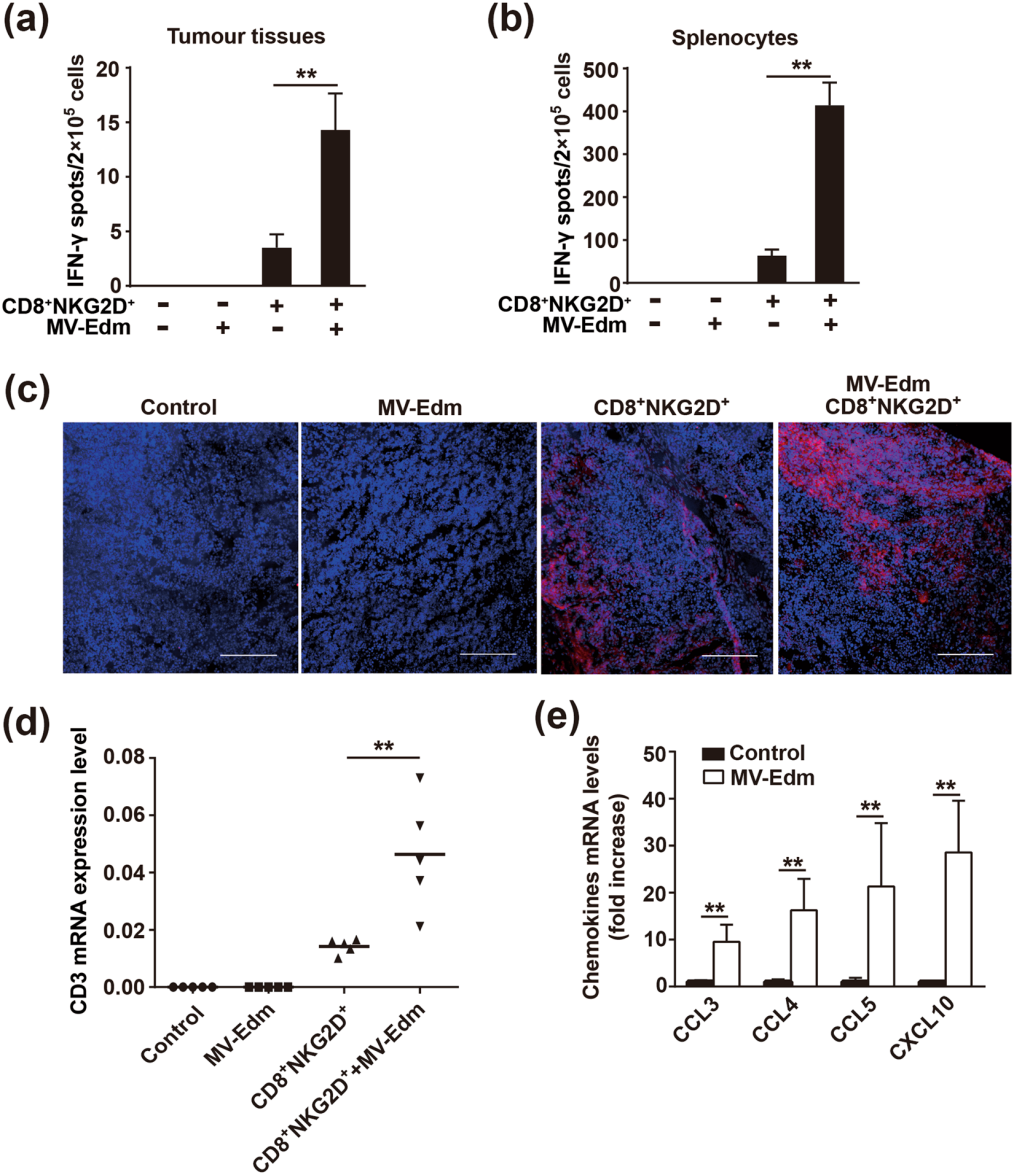
MV-Edm improves activation and tumour infiltration of CD8^+^NKG2D^+^ *in vivo*. Mice bearing subcutaneous HCC were treated with intratumoural injection of MV-Edm (5 × 10^6^ PFU per mouse each injection for 6 times, n = 5), or intravenous injection of CD8^+^NKG2D^+^ cells (1 × 10^7^ per mouse each injection for 6 time, n = 5), or were treated with both (n = 5), or were left untreated (n = 5). 3 days after the last injection of CD8^+^NKG2D^+^ cells, tumours and spleens were dissected. **(a)** Single cell suspensions were obtained from tumours and IFN-γ-producing CD8^+^NKG2D^+^ cells were detected by the human IFN-γ ELISPOT assay kit. Means + SD of 5 mice from each group are shown. **(b)** Single cell suspensions were obtained from the spleens, and then cells were mixed with LM3 cells at a ratio (E:T) of 2:1 for 12 h. The IFN-γ-producing CD8^+^NKG2D^+^ cells were examined by human IFN-γ ELISPOT assay kit. Means + SD of 5 mice each group are shown. **(c)** Cryocuts were obtained from tumour tissues and examined by immunofluorescent staining against human CD3. Representative images are shown for the distribution of CD8^+^NKG2D^+^ cells in tumours. Scale bars are equal to 200 μm. **(d,e)** Total mRNA was obtained from tumour tissues and was quantified by qRT-PCR for the expression levels of **(d)** human CD3, or **(e)** human CCL3, CCL4, CCL5 and CXCL10. Means + SD of 5 mice are shown. * *P* < 0.05, ** *P* < 0.01.

We further investigated whether intratumoural injection of MV-Edm could improve activation of CD8^+^NKG2D^+^ cells. Using Elispot assay, we found that the ratio of IFN-γ-producing CD8^+^NKG2D^+^ cells was increased up to 4 folds within tumour masses (Fig. 4a) and up to 5 folds in the spleens (Fig. 4b) isolated from mice treated with MV-Edm and CD8^+^NKG2D^+^, compared to those receiving CD8^+^NKG2D^+^ alone. In addition to the increased ratio of activated CD8^+^NKG2D^+^ cells within the tumour masses, the number of tumour-infiltrative CD8^+^NKG2D^+^ cells was also significantly increased, as determined by anti-human CD3 staining of tumour tissues (Fig. 4c), and quantified by qRT-PCR for human CD3 expression in tumour tissue (Fig. 4d). As tumour infiltration of lymphocytes is chemokine-dependent, we therefore determined relevant chemokines in HCC tumours. Indeed, chemokines such as CCL3, CCL4, CCL5 and CXCL10 were significantly increased in tumours injected with MV-Edm (Fig. 4e). These data indicate that MV-Edm infection in HCC tumour not only enhances activation but also infiltration of CD8^+^NKG2D^+^ cells.

### MV infection and/or CD8^+^NKG2D^+^ treatment increases IDO1 expression in HCC, and reduction of IDO1 further enhances activation of CD8^+^NKG2D^+^

**Figure 5:**
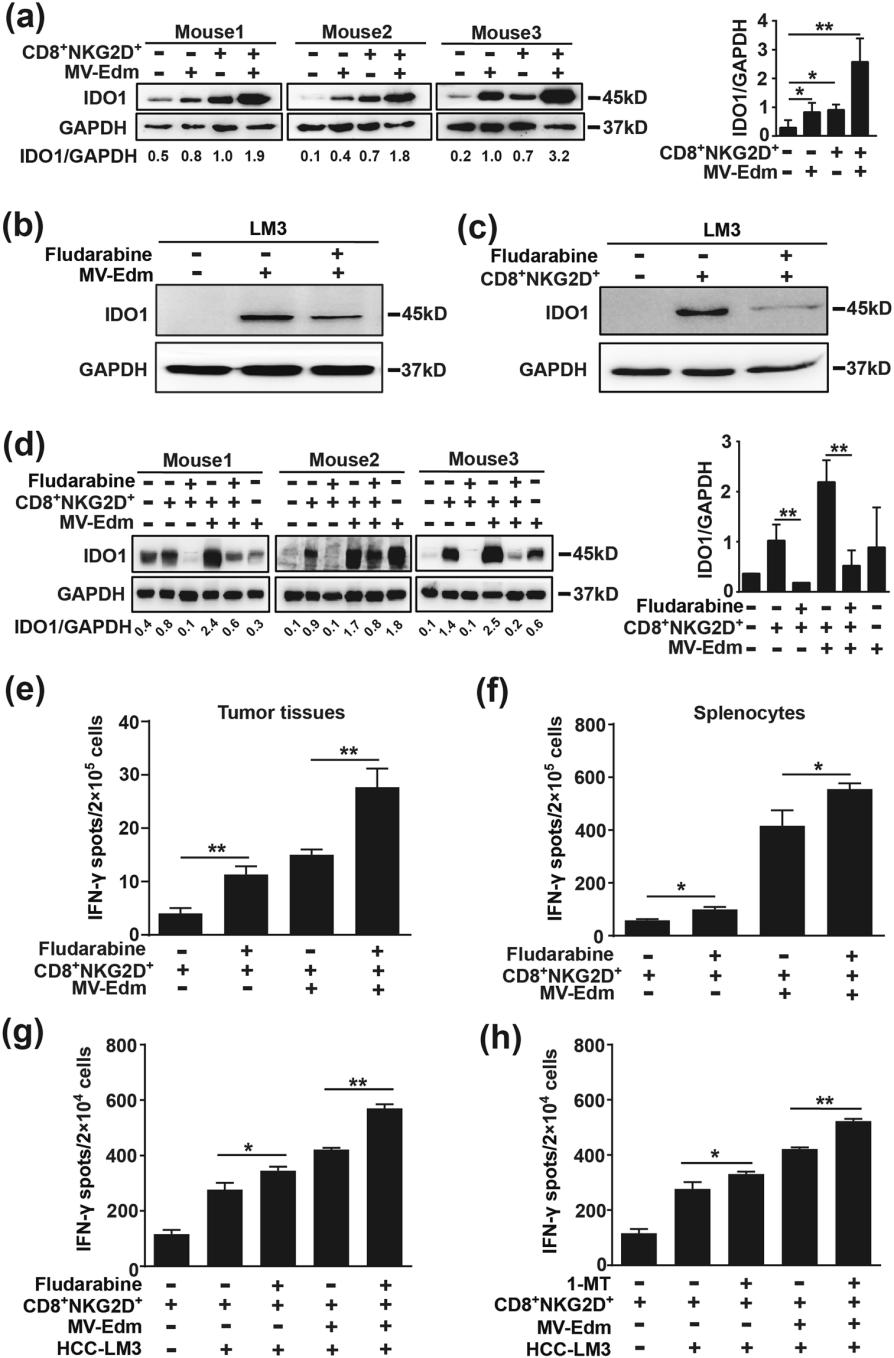
Fludarabine eliminates IDO1 induced by MV-Edm and/or CD8^+^NKG2D^+^ cells leading to improved immune activation. (**a**) Tumour tissues were isolated from mice that received treatments as in Fig. 4. Cell lysates were obtained and IDO1 expression was detected by Western blot analysis. The average IDO1/GAPDH ratio was quantified by densitometric analysis. (**b,c**) LM3 cells were cultured in the presence or absence of 300 nM fludarabine for 24 h, and then **(b)** cells were infected with MV-Edm at a MOI of 1 for another 24 h, or **(c)** cells were treated with CD8^+^NKG2D^+^ cells at the ratio of 2:1 for another 24 h before cell lysates were harvested. Results are representative of three independent experiments. **(d–f)** 1 × 10^7^ LM3 cells were implanted subcutaneously into the flank of Balb/c nude mice. When tumours reached an average volume of 40 mm^3^, mice were randomized to five groups (n = 3 each group). Then mice were received intratumoural injection of MV-Edm (5 × 10^6^ PFU per mouse) on day 0, 1, 7 and were injected intravenously with CD8^+^NKG2D^+^ (1 × 10^7^ per mouse) on day 2 and 8, or injected with CD8^+^NKG2D^+^ cells on day 2 and 8, combined with or without intraperitoneal injection of fludarabine (0.75 mg per mouse) on day 2 and 8. Untreated mice were used as control. 3 days after the last treatment, mice were sacrificed and tumours were dissected. **(d)** Tumour lysates were obtained for IDO1 Western blot. The average IDO1/GAPDH ratio was quantified by densitometric analysis. **(e)** Single cell suspension obtained from tumours or **(f)** spleens was subjected to ELISPOT assay to quantify the human INF-γ-producing cells. Means + SD of three mice are shown. (**g,h**) LM3 cells were cultured in the presence or absence of **(g)** fludarabine (300 nM) or **(h)** 1-MT (100 μM) for 24 h followed by infection with or without MV-Edm (MOI = 1) for another 24 h, then cells were washed and harvested and mixed with CD8^+^NKG2D^+^ cells at a ratio of 2:1 (E:T) for 24 h. The number of INF-γ-producing cells was determined by ELISPOT assay. Means + SD of triplicates from two independent experiments are shown. * *P* < 0.05, ** *P* < 0.01.

Tumours often possess multiple mechanisms to escape immune surveillance. IDO1 is an enzyme that plays crucial role in immunosuppression. IDO1 is often upregulated by IFNs^31^. We then asked if enhanced antitumour immune responses of CD8^+^NKG2D^+^ cells induced by MV-Edm could also upregulate IDO1 expression. Indeed, IDO1 was induced in tumours treated with either viruses or CD8^+^NKG2D^+^ cells alone, and was intensively induced in tumours treated with both (Fig. 5a). We then hypothesized that inhibition of IDO1 would enhance the therapeutic efficacy of MV-Edm and CD8^+^NKG2D^+^ cells. The chemotherapeutic drug fludarabine has been shown to decrease IDO1^27, 32^. We found that both MV-Edm- and CD8^+^NKG2D^+^-induced IDO1 was reduced by fludarabine in HCC cells (Fig. 5b,c). *In vivo*, induced IDO1 could also be markedly decreased by fludarabine (Fig. 5d). We then investigated if IDO1 reduction could further improve activation of adoptively transferred CD8^+^NKG2D^+^ cells. Indeed, the ratio of IFN-γ-producing CD8^+^NKG2D^+^ cells was increased by fludarabine within tumours (Fig. 5e) and in the spleens (Fig. 5 f). In line, fludarabine also improved activation of CD8^+^NKG2D^+^ cells *in vitro* (Fig. 5g). To confirm that the enhanced activation of CD8^+^NKG2D^+^ cells by fludarabine was mediated by reduction of IDO1, we determined the activation of CD8^+^NKG2D^+^ cells in the presence of 1-MT, a specific inhibitor of IDO1. Indeed, IDO1 inhibition by 1-MT *in vitro* achieved a comparable extent of enhanced activation of CD8^+^NKG2D^+^ cells as fludarabine (Fig. 5h). Our findings suggest that reduction of induced IDO1 in HCC enhances the antitumour immune response of CD8^+^NKG2D^+^ cells.

### IDO1 inhibition significantly promotes CD8^+^NKG2D^+^-mediated antitumour efficacy both *in vitro* and *in vivo*

**Figure 6:**
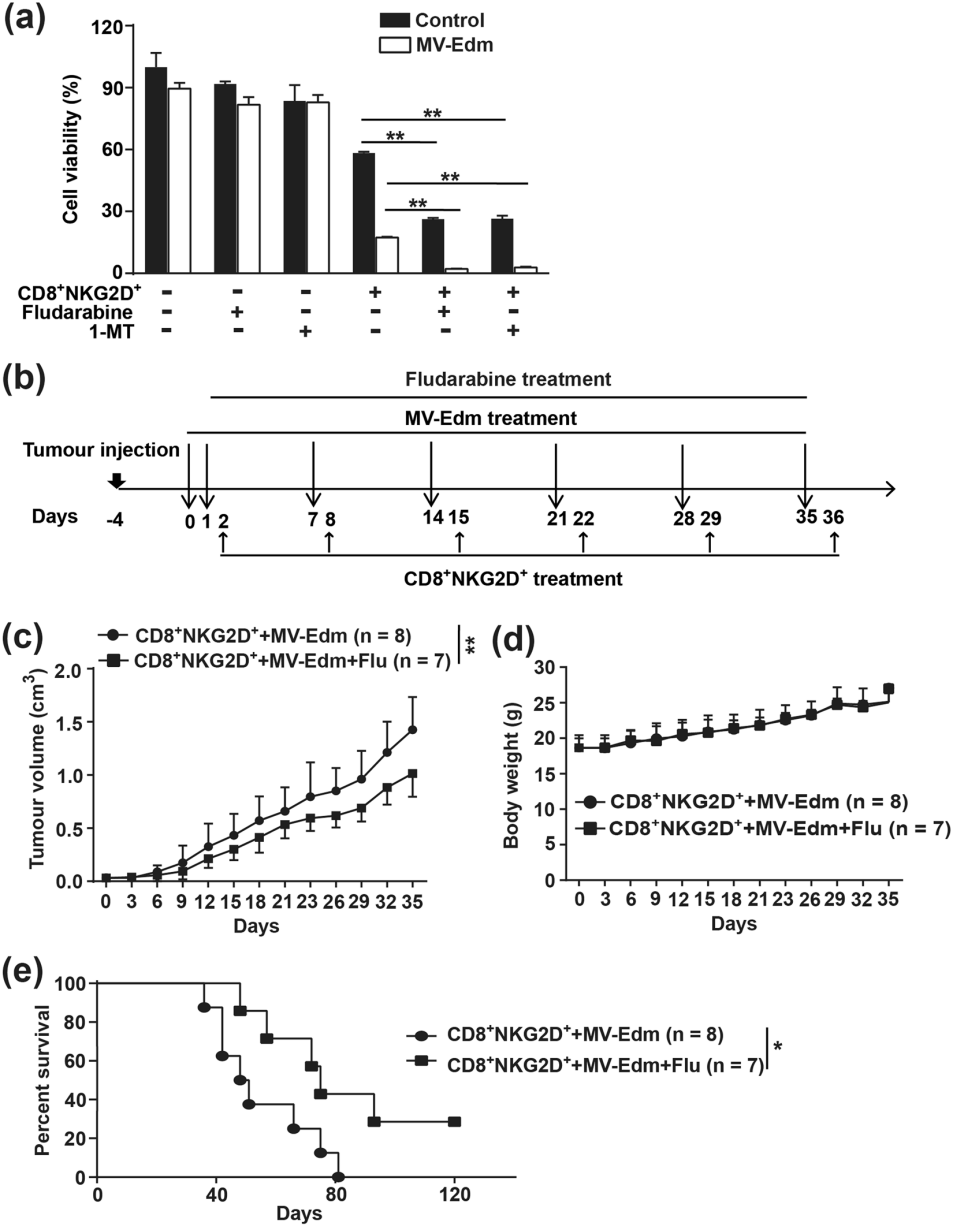
Fludarabine doweregulated IDO1to enhance the antitumour response induced by MV-Edm and CD8^+^NKG2D^+^ cells. (**a**) LM3 cells were cultured in the presence or absence of 300 nM fludarabine or 100 μM 1-MT for 24 h. Cells were infected with MV-Edm (MOI = 1) for another 24 h or were left uninfected. Cells were washed, harvested and mixed with CD8^+^NKG2D^+^ cells at a ratio of 5:1 (E:T) for 24 h. Cell viability was examined by luminescence spectrometry. Means + SD of triplicates are shown. Similar results were obtained in two independent experiments. **(b–e)** 4- to 6-week-old male Balb/c nude mice received subcutaneous injections of 1 × 10^7^ LM3 cells in the right flank. When tumours reached an average volume of 40 mm^3^, mice were randomized to two groups. MV-Edm (5 × 10^6^ PFU each injection) was injected into the tumours on day 0, 1, 7, 14, 21, 28 and 35, followed by intravenous infusion of CD8^+^NKG2D^+^ cells (1 × 10^7^ per mouse), with (filled squares, n = 7) or without (filled circles, n = 7) intraperitoneal injection of fludarabine (0.75 mg per mouse) on day 2, 8, 15, 22, 29 and 36. Mice were sacrificed when tumour volume reached to 2 cm^3^, or when mice appeared moribund. **(b)** The scheme depicts the schedules of the *in vivo* experiment. **(c)** Tumour growth and **(d)** the body weight variation were monitored every 3 days. Means + SD are shown. **(e)** Survival was determined and plotted for Kaplan-Meier survival analysis and analyzed by log-rank test. * *P* < 0.05, ** *P* < 0.01.

Next we wanted to know if improved activation of CD8^+^NKG2D^+^ cells by fludarabine could achieve enhanced antitumour efficacy. Again, both fludarabine and 1-MT enhanced CD8^+^NKG2D^+^-mediated cytotoxicity against HCC cells *in vitro* (Fig. 6a). Then we compared the antitumour efficacy of MV-Edm combined with CD8^+^NKG2D^+^ cells in the presence or absence of fludarabine (Fig. 6b). Growth inhibition of HCC was achieved in the presence of fludarabine (Fig. 6c) and no obvious side-effect was observed in both treated mice as monitored by body weight (Fig. 6d). Survival was prolonged in mice that received fludarabine (Fig. 6e). These data show that inhibition of induced IDO1 by fludarabine further enhances efficacies of MV-Edm combined with CD8^+^NKG2D^+^ cells for the treatment of HCC.

## Discussion

The mechanisms and parameters underlying MV-Edm-elicited antitumour immunity remain to be clarified. In this study, we showed that MV-Edm-infected HCC improved infiltration and *in situ* activation of adoptively transferred CD8^+^NKG2D^+^ cells, leading to enhanced oncolysis. However, MV-Edm and/or adoptive transfer of CD8^+^NKG2D^+^ also massively induced the immune suppressive enzyme IDO1 in HCC. When IDO1 was reduced by fludarabine, enhancement of antitumour efficacy was achieved. We thus provide a novel strategy for the therapy of HCC, and show for the first time that inducible immune suppression must be considered and can be targeted during therapy by means of viro-immunotherapy.

Because of the lack of good immune competent mouse models of MV-Edm in eliciting antitumour immune responses, we utilized adoptive transfer of CD8^+^NKG2D^+^ cells in nude mice bearing human HCC. CD8^+^NKG2D^+^ cell is a subpopulation of CIK cells that can be directly activated by MICA/B, the specific ligands for NKG2D, expressed on HCC cells. In our experimental setting, the majority (over 90%) of CIK cells expressed NKG2D, which can be activated independent of MHC molecules. Thus, our results reflect the capabilities of MV-Edm in activating innate immunity against HCC. The exact role of MV-Edm in eliciting antitumour adaptive immune responses, however, require further investigation in primate models or clinical trials. It has been shown *ex vivo* that MV-infected mesothelioma cells promote maturation of dendritic cells (DCs) inducing proliferation of tumour-specific T cells^17^. Here, we complement these findings by showing that MV-infected HCC cells undergo ICD (supplemental data), an indispensable process required for activation of DCs. These observations indicate that MV may also activate adaptive antitumour immunity.

Genetically engineered adenoviruses combined with adoptive CIK or T cells have shown enhanced antitumour efficacy in several preclinical solid tumour models^33–35^. However, the underlying mechanisms remain to be elucidated. In this study, we show for the first time that the enhanced antitumour responses of CD8^+^NKG2D^+^ in MV-Edm-infected HCC cells were contributed by two ways. First, MV-Edm infection stimulated HCC cells to express high-level of stress-inducible MICA/B, in turn augmenting the activation of CD8^+^NKG2D^+^ cells. The activated CD8^+^NKG2D^+^ cells may secrete perforin and granzyme to trigger cancer cell death. In line, another study shows that doxycycline enhances susceptibility of cancer cells to CIK cells by increasing MICA/B membrane expression^36^. Second, FasL was upregulated in CD8^+^NKG2D^+^ cells co-cultured with MV-Edm-infected HCC cells, subsequently enhancing apoptosis via interaction with its cognate receptor Fas expressed in cancer cells^37^. However, we did not observe an increase of Fas in cancer cells after MV-Edm infection. It has been shown that FasL delivery to the cell surface is controlled by polarized degranulation in NK cells or T cells^38^. As we have shown that CD8^+^NKG2D^+^ cells can be activated by MICA/B, which are upregulated in MV-Edm-infected HCC cells, we speculate that the upreulation of FasL might be the consequence of enhanced degranulation in activated CD8^+^NKG2D^+^ cells. It has been described that oncolytic vesicular stomatitis virus infection promotes immune evasion by preventing NKG2D-ligand expression^39^. However, some viruses, including human cytomegalovirus (HCMV) and adenovirus, induce NKG2D-ligand expression, which could sensitize tumour cells to NKG2D-depends NK cell lysis and lead to tumour rejection^40, 41^. Thus, it is crucial to identify the specificity of different OVs concerning virus-mediated immune modulation.

Beyond enhanced activation of CD8^+^NKG2D^+^ cells by MV-Edm-infected HCC cells both *in vitro* and *in vivo*, we found that intratumoural administration of MV-Edm also increased infiltration of intravenously transferred CD8^+^NKG2D^+^ cells. This was mediated by upregulated chemokines within the tumour after MV-Edm infection. In line, a recent study showed that intratumoural injection of HSV-2 increased infiltration of adoptive T cells by elevated chemokines^42^. This makes the strategy combining OVs with adoptive CD8^+^NKG2D^+^ cells extremely attractive for solid tumour treatment, as enhanced chemoattraction of adoptive cells (via induction of chemokines) and *in situ* activation (via upregulation of MICA/B) work hand-in-hand to improve therapeutic efficacy.

In addition to an enhanced antitumour effect, we found that MV-Edm combined with adoptive transfer of CD8^+^NKG2D^+^ cells almost completely inhibited metastases. This effect is mainly contributed by CD8^+^NKG2D^+^ cells, since adoptive transfer alone resulted in a similar decrease of metastases. Thus, adoptive CIK cells may be well suited for controlling metastases^20^.

Upregulation of indoleamine 2,3-dioxygenase 1 (IDO1) significantly impairs activation and proliferation of adoptive T cell^43, 44^. IDO1 can be reactively induced in response to inflammation in either IFN-γ-dependent or -independent manner^28^. In agreement with this, immuosuppressive IDO1 was also massively induced in HCC intratumourally injected with MV-Edm combined with intravenous transfer of CD8^+^NKG2D^+^ cells. In addition, both MV-Edm infection or CD8^+^NKG2D^+^ transfer alone also induced IDO1, however, to a lesser extent. The increase of IDO1 expression in HCC is probably induced either by the inflammation stimulated by viral infection, or by immune activation of CD8^+^NKG2D^+^ cells. Induced IDO1 compromised MV-Edm-enhanced immune activation and antitumour efficacy of CD8^+^NKG2D^+^ cells, and the IDO1 inhibitor fludarabine, a drug known to decrease IDO1 expression^32^, achieved a more profound antitumour immune responses against HCC cells *in vivo*. Of note, fludarabine alone exerted no cytotoxicity on HCC cells shown in Fig. 6a, however, was sufficient to enhance susceptibility of cancer cells to CD8^+^NKG2D^+^ cells. In tumour-bearing mice, fludarabine also effectively decreased IDO1 in HCC induced by MV-Edm and adoptive CD8^+^NKG2D^+^ cells, leading to an enhanced antitumour immune responses and prolonged survival. Again, the low-dose of fludarabine used in this study was insufficient to inhibit HCC cell growth, or to promote cell death *in vivo* (unpublished). Our study raises a critical point in viro-immunotherapy, i.e. how to counteract therapy-induced immune escape of cancer.

In conclusion, our study shows that local administration of MV-Edm improves antitumour activity of adoptive CD8^+^NKG2D^+^ cells in HCC. We also delineate the mechanisms underlying the enhanced innate immune activation and, importantly, the potential improvement of viro-immunotherapy by targeting therapy-induced immune suppression. Given that both MV-Edm and adoptive transfer of CIK cells are being investigated in clinical trials, our therapeutic strategy could be readily translated to optimize therapeutic outcomes. Furthermore, drug fludarabine should be considered for further therapeutic improvement in viro-immunotherapy against cancer.

## Methods

### Cell lines

Human hepatocellular carcinoma cell lines HCC-LM3 (LM3) and MHCC-97H (97H) were obtained from Live Cancer Institute of Zhongshan Hospotal (Shanghai, China)^45, 46^. Vero African green monkey kidney cell was obtained from American Type Culture Collection (Manassas, VA). Cells were cultured in Dulbecco’s modified Eagle’s medium (DMEM) supplemented with 10% fetal bovine serum, 100 U/L penicillin and 0.1 mg/ml streptomycin (all from Life Technologies) and maintained in a humidified incubator with 5% CO_2_ at 37 °C.

### Viruses

Measles virus Edmonston vaccine lineage seed B (MV-Edm, kindly provided by S. Russell, Mayo Clinic) was propagated in Vero cells with a multiplicity of infection (MOI) of 0.01 at 37 °C for 2 days. Cells were harvested, and viral particles were released by three cycles of snap-freezing in liquid nitrogen and thawing in a 37 °C water bath. Viral titers were determined by 50% endpoint dilution assays (50% tissue culture infective dose [TICD_50_]) on Vero cells.

### Generation of CD8^+^NKG2D^+^ cells

Human peripheral blood lymphocytes (PBMCs) were isolated from healthy human volunteers, and the informed consents were obtained from all volunteers. Cells were cultured in medium consisting of GT-T551 (TaKaRa) supplemented with 2000 IU/ml recombinant human interferon-gamma (Chemo Wanbang Biopharma, Shanghai, China), 10 mg/ml OK432 (Chugai Pharmaceutical, Chome, Japan), 2% autologous plasma and 1% penicillin-streptomycin on day 1 for 24 h. Then cells were transferred to an anti-CD3 (T&L Biological Technology, Beijing, China) antibody-coated flask in GT-T551 medium supplemented with 700 IU/ml recombinant human interleukin-2 (IL-2) (BD Biosciences) for 14 days. Cells were harvested and stored at −80 °C for further experiments. The protocol of this study was approved by the research ethics committee of Medical School of Nanjing University. The experimental methods were carried out in accordance with the approved guidelines and regulations.

### Flow cytometry

For analysis of CD8^+^NKG2D^+^ cells, cells were harvested and stained with CD3-FITC, CD8-PE, NKG2D-APC (all from BD Biosciences). HCC cells were infected with or without MV-Edm (MOI = 1) for 24 h, then cells were either harvested and stained with antibodies against MICA/B-PE or Fas-FITC (BD Biosciences), or CD8^+^NKG2D^+^ cells for another 24 h, then cells were harvested and stained with antibodies against CD3-FITC and CD95L (BD Biosciences). The incubation of antibodies was processed on ice for 30 min. Flow cytometry analysis was carried out by FACSCalibur instrument (BD). The data was analyzed using FlowJo software (Tree Star Inc., Ashland, Oregon).

### CD8^+^NKG2D^+^ cells cytotoxicity assay

LM3 and 97H cells stably expressing luciferase were used as target cells for cytotoxicity assay of CD8^+^NKG2D^+^ cells. Target cells were plated into 96-well plates (1 × 10^4^ cells/well). The effecter CD8^+^NKG2D^+^ cells were added at various ratios (effector: target, E:T). After 8 h, CD8^+^NKG2D^+^ cells were removed and the wells were rinsed with PBS. The viability of target cells was determined by luciferase activity using Luciferase assay system according to manufacturer’s instructions (Promega).

Or LM3 cells were cultured in the presence or absence of fludarabine (TCI, Tokyo, Japan, 300 nM) or 1-MT (Sigma, 100 μM) for 24 h followed by infection with or without MV-Edm (MOI = 1) for another 24 h, cells were harvested and plated into 96-well plates (1 × 10^4^ cells/well), then CD8^+^NKG2D^+^ cells were added at a ratio of 2:1 (E:T) for 24 h. Cell viability was determined as described above.

### Western blot

Cells were pelleted and lysed using lysis buffer (Roche). The protein concentration was determined. The samples were migrated on SDS-PAGE and transferred onto PVDF membranes (Roche). After blocking with 5% nonfat milk, the membrane was incubated with primary antibodies followed by incubation with horseradish peroxidase-conjugated secondary antibodies. Signals were detected using an enhanced chemiluminescence reagent (Millipore) and subjected to the Alpha Innotech Fluor Chem-FC2 imaging system (Alpha Innotech, San Leandro, CA). Antibodies were as follows: anti-GAPDH (Bioworld, Nanjing, China, 1:5000 diluted), anti-Caspase-3 (Cell Signaling Technology, 1:1000 diluted), anti-IDO-1 (Abcam, 1:500 diluted).

### Quantitative PCR

RNA was extracted with TRIzol (Life Technology), and was reverse-transcribed using the synthesis system (TaKaRa). Then SYBR green PCR master mix (Applied Biosystems) reagent was used according to the manufacturer’s protocol and the samples was analyzed using a Real-Time PCR system (ABI 7300). Primers used in this experiment were: GAPDH forward 5′-CCACCCATGGCAAATTCCATGGCA-3′ and reverse 5′-TCTAGACGGCAGGTCAGGTCCACC-3′; CCL3 forward 5′-AGTTCTCTGCATC ACTTGCTG-3′ and reverse 5′-CGGCTTCGCTTGGTTAGGAA-3′; CCL4 forward 5′-CTGTGCTGATCCCAGTGAATC-3′ and reverse 5′-TCAGTTCAGTTCCAGGT CATACA-3′; CCL5 forward 5′-CATATGGCTCGGACACCACTC-3′ and reverse 5′-CGACTGCAAGATTGGAGCAC-3′; CXCL10 forward 5′-CTTCCAAGGATGGAC CACACA-3′ and reverse 5′-CCTTCCTACAGGAGTAGTAGCAG-3′; CD3 forward 5′-GCCAGAACCAGCTCTATAAC-3′ and reverse 5′-TAGGCCTCCGCCATCTTA TC-3′.

### IFN-γ Enzyme-Linked Immunosorbent Spot (ELISPOT) assay

The secreted IFN-γ from activated CD8^+^NKG2D^+^ cells was detected by the human IFN-γ ELISPOT Kit (Mabtech) according to the manufacturer’s protocol. Briefly, *in vitro*, CD8^+^NKG2D^+^ and HCC cells were mixed at a ratio of 2:1 (E:T) and seeded in 96-well plate (1 × 10^4^ HCC cells/well) coated with IFN-γ capture antibody. 12 or 24 h later, cells were removed and the wells were rinsed with PBS. The biotinylated detection antibody against IFN-γ was added and the plate was incubated for 2 h at room temperature. Then the wells were rinsed with PBS followed by incubation with Streptoavidin-APL for 1 h at room temperature. The substrate BCIP/NBT-plus was added until spots emerged. The reaction was stopped by washes with tap water. Or cells isolated from tumour masses were seeded in IFN-γ capture antibody-coated 96-well plate at a density of 2 × 10^5^ cells/well for 12 h. Or cells isolated from spleens were mixed with LM3 cells at the ratio of 2:1 (E:T) and then seeded in IFN-γ capture antibody-coated 96-well plate at a density of 2 × 10^5^ cells/well for 12 h. The secreted IFN-γ was then determined as described above.

### *In vivo* experiments and tumour models

Male Balb/c nude mice (4–6 week old) were purchased from the Model Animal Research Center of Nanjing University (Nanjing, China), and were maintained under specific pathogen-free conditions. For xenograft studies, mice were injected subcutaneously with 1 × 10^7^ LM3 cells in the right flank. When tumour volumes reached to an average of 40 mm^3^, the treatments were started.

HCC-bearing mice were randomized to 4 groups based on tumour sizes so that the average tumour volumes of each group are comparable prior to treatment. One group was treated with intratumoural injection of MV-Edm (5 × 10^6^ PFU/mouse), one received intravenous injection of CD8^+^NKG2D^+^ cells (10^7^ cells/mouse), one received both MV-Edm and CD8^+^NKG2D^+^ cells. Untreated group was used as control. Tumour volumes were monitored twice a week by caliper measurement (volume = length × width^2^/2). The body weight and behaviors were monitored in parallel. Mice were euthanized when tumour volumes reached to 2 cm^3^, or when mice appeared moribund.

To evaluate the expression and role of IDO1 *in vivo*, LM3-bearing mice were randomized according to the tumour sizes to five groups (n = 3 each group). Then mice were received intratumoural injection of MV-Edm (5 × 10^6^ PFU/mouse) on day 0, 1, 7 and were injected with CD8^+^NKG2D^+^ cells via tail vein (1 × 10^7^ cells/mouse per mouse) on day 2 and 8, or injected with CD8^+^NKG2D^+^ cells via tail vein (1 × 10^7^ cells/mouse) on day 2 and 8, combined with or without intraperitoneal injection of fludarabine (0.75 mg/mouse). Three days after the last treatment, mice were sacrificed and tumours were dissected.

To evaluate the influence of IDO1 in the combinatorial therapy of MV-Edm and CD8^+^NKG2D^+^ cells, LM3- bearing mice were randomly divided into two groups based on tumour sizes so that the average tumour volumes of each group are comparable prior to treatment: both groups received intratumoural injection of MV-Edm (5 × 10^6^ PFU/mouse) and intravenous injection of CD8^+^NKG2D^+^ cells (1 × 10^7^ cells/mouse), and one group received additional intraperitoneal injection of fludarabine (0.75 mg/mouse). Tumour volumes, body weight and behaviors of mice were monitored as described above.

All animal experimental procedures were approved by the Animal Care Committee of Nanjing University in accordance with Institutional Animal Care and Use Committee guidelines.

### Immunofluorescent staining

Tumour tissues were dissected, embedded in tissue freezing medium, and frozen sectioned using freezing microtome (Lecia CM1900, Germany). 10 μm sections fixed in acetone were rinsed with PBS, and were blocked with 2% BSA followed by incubation with rabbit-anti-CD3 (Abcam, 1:100 diluted) at 4 °C overnight. Then sections were incubated with anti-rabbit secondary antibody (Invitrogen, 1:200 diluted) for 1 h at 37 °C. Nuclei were stained with 1 μg/ml DAPI (Sigma). The images were captured using a confocal fluorescence microscope (Olympus) and were analyzed by using FV10-ASW software.

### Statistical analysis

GraphPad software (Prism, 5.0) and Microsoft Excel was used to plot and analyze all the graphs. Student’s t test was used for most statistical analyses. Statistical analysis of tumour area among the groups was done using repeated measures ANOVA Statistical analysis of survival curves was performed using Log rank (Mantel-Cox) Test.

## Electronic supplementary material

Supplementary Information
