# Supplementary material for: Oncolytic measles virus enhances antitumour responses of adoptive CD8+NKG2D+ cells in hepatocellular carcinoma treatment
Source: Sci Rep. 2017 Jul 12;7:5170. doi: 10.1038/s41598-017-05500-z (PMC5507973; doi:10.1038/s41598-017-05500-z)
Supplement: Supplementary file 1 — Supplementary Information [file 41598_2017_5500_MOESM1_ESM.pdf]

Oncolytic measles virus enhances antitumour responses of adoptive CD8$^+$NKG2D$^+$ cells in hepatocellular carcinoma treatment

Aiping Chen[1,2,4], Yonghui Zhang[1,4], Gang Meng[1,2], Dengxu Jiang[1], Hailin Zhang[1], Meihong Zheng[1], Mao Xia[1], Aiqin Jiang[1], Junhua Wu[1*], Christian Beltinger[3], and Jiwu Wei[1,2*]

(Supplementary Information)

This file contains

1. Supplementary Figure S1
2. Figure Legend for Supplementary Figure S1
3. Supplementary Methods

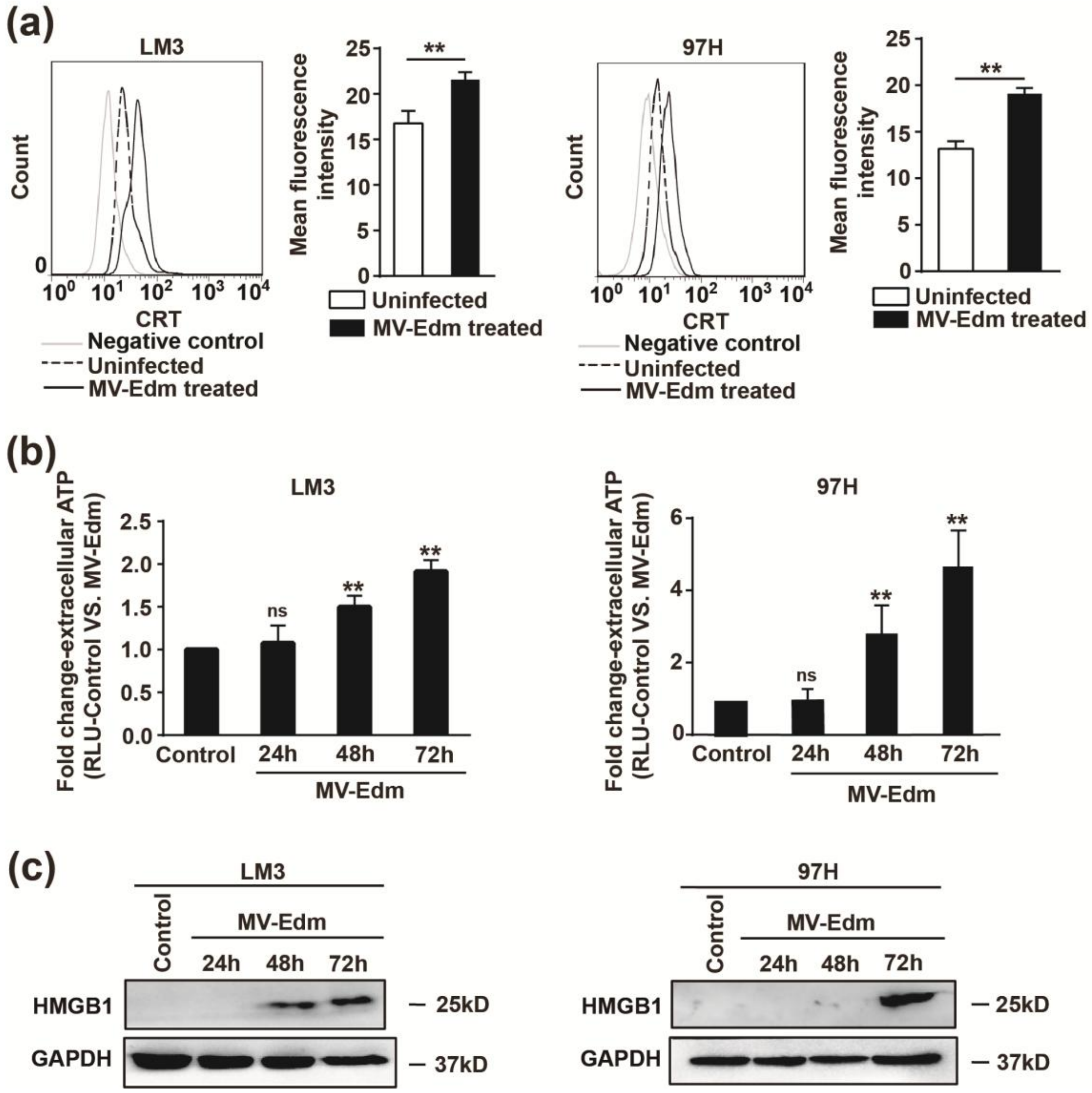
(a)
LM3
Count
0
10^0 10^1 10^2 10^3 10^4
CRT
Negative control
Uninfected
MV-Edm treated
Mean fluorescence intensity
25
20
15
10
5
0
**
Uninfected
MV-Edm treated
97H
Count
CRT
Negative control
Uninfected
MV-Edm treated
Mean fluorescence intensity
**
Uninfected
MV-Edm treated
(b)
LM3
Fold change-extracellular ATP (RLU-Control VS. MV-Edm)
2.5
2.0
1.5
1.0
0.5
0.0
ns
**
**
Control
24h
48h
72h
MV-Edm
97H
Fold change-extracellular ATP (RLU-Control VS. MV-Edm)
6
4
2
0
ns
**
**
Control
24h
48h
72h
MV-Edm
(c)
LM3
Control
MV-Edm
24h
48h
72h
HMGB1
GAPDH
25kD
37kD
97H
Control
MV-Edm
24h
48h
72h
HMGB1
GAPDH
25kD
37kD

Figure legends:

Supplementary Figure 1. MV-Edm induces immunogenic cell death in HCC cells. **(a)** LM3 and 97H cells were infected with MV-Edm (MOI = 1) for 24 h, then cells were harvested and stained by anti-CRT-PE before subjected to flow cytometry. Unstained cells were used as negative controls. An overlay of histograms representative of 3 independent experiments, and the mean fluorescence intensity of CRT averaged from 3 independent experiments are shown. **(b, c)** LM3 and 97H cells were infected with MV-Edm (MOI = 1) for 24, 48 and 72 h, respectively. Uninfected HCC cells were used as controls. Supernatants were then harvested. **(b)** Extracellular ATP levels were quantified by a luciferase based ATP kit. Means + SD of three independent experiments are shown. **(c)** HMGB1 in supernatants was examined by western blot. The representative blots from three independent experiments are shown. *ns*, not significant, ***** $P < 0.05$ and ****** $P < 0.01$.

Supplementary Methods

Flow cytometry. HCC cells were grown on 12-well plates and infected with or without MV-Edm (MOI = 1) for 24 h. Then cells were harvested and stained with anti-CRT-PE (Abcam) for 30 min at 4 ℃. Flow cytometry analysis was carried out by FACSCalibur instrument (BD) and data was analyzed using FlowJo software (Tree Star Inc.).

ATP assay. HCC cells were grown on 12-well plates and infected with or without MV-Edm (MOI = 1) for 24, 48 and 72 h, respectively. The Supernatants were then

harvested by centrifugation at 12000 g for 10 min at 4 ℃. ATP was measured by ATP Assay Kit (Beyotime, China) according to the manufacturer’s protocol.

Western blot. HCC cells were grown on 12-well plates and infected with or without MV-Edm (MOI = 1) for 24, 48 and 72 h, respectively. Then the supernatants were harvested. To assess the HMGB1 release, 2 ml supernatant was concentrated by centrifugation via Amicon 3K Ultra-4 centrifugal filter units (Millipore) at 4,300 rpm for 20 min. Then total protein harvested from supernatant was separated by SDS-PAGE and transferred onto PVDF membranes (Roche). After blocking with 5% non-fat milk in Tris-buffered saline containing 0.1% Tween 20, the membrane was incubated with anti-HMGB1 (Abcam, 1:1,000 diluted) followed by incubation with appropriate horseradish peroxidase-conjugated secondary antibodies. Signals were detected using an enhanced chemiluminescence reagent (Millipore) and subjected to the Alpha Innotech Fluor Chem-FC2 imaging system (Alpha Innotech).
